# MicroRNA-146a rs2910164 is associated with severe preeclampsia in Black South African women on HAART

**DOI:** 10.1186/s12863-016-0469-z

**Published:** 2017-01-19

**Authors:** Niren Ray Maharaj, Prithiksha Ramkaran, Siddharthiya Pillay, Anil Amichund Chuturgoon

**Affiliations:** 1 Department of Obstetrics and Gynaecology, Prince Mshiyeni Memorial Hospital, Durban, South Africa; 2 Discipline of Medical Biochemistry and Chemical Pathology, School of Laboratory Medicine and Medical Sciences, College of Health Sciences, University of KwaZulu-Natal, Howard College Campus, George Campbell Building—South Entrance, 3rd Floor, King George V Avenue, Durban, South Africa

**Keywords:** miR-146a, rs2910164, Preeclampsia, HIV, HAART, Black SA women

## Abstract

**Background:**

South African (SA) Black women have a high prevalence of preeclampsia and HIV, both conditions associated with increased inflammation. miR-146a is an inflammatory-associated miR and a common single nucleotide polymorphism (rs2910164) has been associated with several disease conditions. To date, this SNP has not been investigated in SA Black women. We therefore aimed to investigate the miR-146a G > C SNP in SA Blacks with preeclampsia, and further examine possible association among preeclamptic (PE) women with HIV infection on HAART.

**Methods:**

This hospital-based, case-control study included 95 normotensive and 98 PE Black SA women (aged 16–46 years old). Patients and controls were genotyped by PCR-RFLP. Using a Cytometric Bead Array assay, serum cytokine levels (including Th1- and Th2-related cytokines) were determined in 4 groups of pregnant women, viz: normotensive, HIV infected, PE + HIV infected, and PE women.

**Results:**

There was no significant association between the miR-146a polymorphism and PE susceptibility in our data. However, in the subgroup analyses, the variant genotypes (GC/CC) were significantly associated with lower severe PE risk (*p* = 0.0497), more especially in the presence of HIV and HAART (*p* = 0.017). In the normotensive group, the variant genotypes were associated with lower IL-2 in both the total normotensive group (269 ± 1.26 (36) vs 273 ± 1.31 (23); *p* = 0.035) and the PE HIV+ sub-group 265 ± 1.54 (19) vs 271 ± 1.38 (11); *p =* 0.008).

**Conclusions:**

Our study suggests that miR-146a rs2910164 polymorphism might not be associated with PE susceptibility, cytokines or related features. However, the miR-146a GC/CC genotype might reduce susceptibility to severe PE, which might be further influenced by the presence of co-morbid HIV infection among pregnant women on HAART. This variant genotype may also be associated with reduced circulating IL-2 levels and thus reduced pro-inflammatory response in normotensive women, which may be further influenced by the presence of HIV infection and HAART.

## Background

Preeclampsia (PE) and HIV infection contribute significantly to adverse maternal and perinatal outcomes globally [1, 2]. In developing countries such as South Africa, the prevalence of both conditions remains high and these co-morbidities are important causes of mortality. Unfortunately, there is a paucity of data on the relationship between PE and HIV co-infection both in developed and developing countries [3–5].

Preeclampsia is heterogeneous in nature, and is associated with differences in the timing of disease, clinical manifestations, severity of organ damage, maternal and foetal outcomes and complications. The diverse nature of PE is further evident in the severity in which it can manifest i.e. mild and severe disease [6]. Although the definition of severe PE varies, it is generally associated inter alia with markedly elevated blood pressure, maternal neurological complications, seizures, signs of hepatic and renal dysfunction, and foetal affectation [7]. In developing countries, severe forms of PE and eclampsia are more common, and range from a low of 4% of all deliveries to 18% in parts of Africa [8].

Preeclampsia is associated with a pro-inflammatory milieu, in which cytokines play a significant role as mediators [9]. Whilst normal pregnancy also elicits an inflammatory response, PE is characterised by an excessive inflammatory response [10], and so it is important to look at the regulation of the inflammatory cytokine mediators. A Th1/Th2 cell ratio imbalance has been hypothesised, skewing towards the Th1 response [11], demonstrated by Toldi et al. 2011 [12]. Th1 cells are involved in pro-inflammatory cytokine production and cell-mediated immunity; Th2 cells are involved in anti-inflammatory cytokine and immune-suppressor cytokine production and B-cell humoral immunity [10].

Other pathogenic mechanisms such as immune maladaptation, inadequate placental development and trophoblast invasion, placental ischaemia, oxidative stress and thrombosis are all thought to represent key factors in the development of PE [13]. All of these components have genetic factors that may be involved in the pathogenesis [13], which may alter susceptibility to preeclampsia or its complications. The genetic basis of PE is further supported by epidemiological findings that show a 2–5 fold increased risk of PE in women with a maternal history of this disease [14].

MicroRNAs (miRNAs; miRs) are small (±22 nucleotides), endogenous, non-coding RNA’s that regulate gene expression by modulating the expression of multiple target mRNAs, inducing either translational inhibition or mRNA degradation [15]. They are involved in various pathological processes [15], including the regulation of innate and adaptive immune responses [16]. MiRNA-146a is involved in modulating the negative regulation of Toll like receptor (TLR) signalling, and inflammatory cytokines [17]. MiRNA-146a lies at the crossroads of biological processes that involve innate-immune responses, viral-infection and inflammatory disease [17]. It prevents an overstimulation of the inflammatory response through its recognised target genes, IRAK-1 and TRAF-6 [16, 18], and its dysregulation is associated with many inflammatory diseases including rheumatoid arthritis (RA) and systemic lupus erythematosus (SLE) [15]. Its role in preeclampsia has not been established.

Polymorphisms may affect miRNA expression, maturation or mRNA recognition, and alter disease susceptibility [15]. A common miRNA-146a single nucleotide polymorphism (SNP) (rs2910164) is located within the seed sequence of pre-miRNA 146a, which is the miRNA-146a precursor [19, 20]. This functional polymorphism has been associated with various inflammatory diseases including RA and SLE [21, 22].

Based on the inflammatory milieu in PE and HIV, the regulatory role of miRNA-146a in inflammatory responses, and the association of miRNA-146a rs 2910164 with inflammatory diseases, this study investigated the role of rs2910164 in PE. Due to the high prevalence of HIV, we included women with HIV infection on highly active antiretroviral therapy (HAART) in order to identify a possible differential influence [4]. HAART is a standard treatment consisting of a combination of at least three drugs. In the present study, efavirenz, emtracitabine, and tenofovir were used. To the best of our knowledge, this is the first investigation in high risk Black SA women during pregnancy.

## Methods

### Study population and sample collection

Institutional ethical and hospital regulatory permission was obtained for the study (Biomedical Research Ethics Committee, University of KwaZulu-Natal, South Africa; reference number BE119/11). After informed consent was obtained, participants were recruited over a 14-month period (from July 2013 to September 2014) at the maternity unit at Prince Mshiyeni Memorial Hospital, Durban, South Africa. This hospital is a regional level facility and serves a predominantly semi-urban African population. Normotensive [*n* = 95, (80 genotyped) age range: 16–46 years] and PE patients [*n* = 98, (60 genotyped) age range: 16–42 years] were enrolled into the study. To maintain ethnographic and anthropometric consistency, all patients recruited were of African descent, resident in the same geographical location and of Zulu ethnicity. All patients were non-smokers, non-consumers of alcohol or recreational drugs, and all HIV infected patients were on HAART (tenofovir, emtricitabine, efavirenz) as per the National guidelines [23]. Calcium supplementation was administered routinely to all patients attending the clinic. Women with gestational hypertension, renal disease, diabetes mellitus, chronic hypertension and collagen vascular disease were excluded from this study. Preeclampsia was defined as a blood pressure ≥140mmHg systolic or ≥ 90mmHg diastolic on two occasions at least 4 h apart after 20 weeks of gestation in a woman with previously normal blood pressure (<140mmHg systolic, >90mmHg diastolic), consistent with guidelines [7]. All patients had proteinuria ≥1 on urine dipstick testing. Data on all patients was obtained from the institution’s maternity case records and laboratory data from the National Health Laboratory Services^®^ computerised database at the institution. HIV was diagnosed on a rapid test kit and weight was categorised as: normal weight (BMI: 18–25), overweight (BMI: 25–30), obese (BMI: 30+). Early onset PE was considered as ≤34 weeks of gestation [24]. Severe PE was diagnosed when features included any of the following: systolic blood pressure ≥160mmHg or diastolic blood pressure ≥110mmHg; maternal neurological disorders such as persistent headaches and brisk reflexes, eclampsia, acute pulmonary oedema, proteinuria ≥5g/day, oliguria <500cc/day, creatinine >120μmol/L, features HELLP syndrome and thrombocytopenia <100,000/mm^3^, foetal criteria including intrauterine growth retardation, oligohydramnios, or foetal death in utero [6, 7].

### Cytokine quantification

Since miR-146a is an inflammatory associated miRNA, we decided to measure the levels of certain cytokines. The BD Cytometric Bead Array Human Th1/Th2/Th17 Cytokine kit was used to measure Interleukin (IL)-2, IL-4, IL-6, IL-10, IL17a and tumour necrosis factor-alpha (TNF-α) protein levels in a serum samples. Briefly, lyophilized standards were prepared by reconstitution and serial dilution (1:2–1:256) in assay diluent immediately before staining with Capture Beads and Phycoerythrin Detection Reagent. All serum samples were also diluted in assay diluent (1:4) before staining with Capture Beads and Phycoerythrin Detection Reagent. For the staining procedure, 50 μL of each standard and unknown sample was added to appropriately labelled sample tubes followed by 50 μL of the Human Th1/Th2/Th17 Phycoerythrin Detection Reagent and incubated (3 h, RT, protected from light). Following incubation 1 mL of Wash Buffer was added to each assay tube and centrifuged at 200g for 5 min. The supernatant from each assay tube was then carefully aspirated and 300 μL of Wash Buffer was added to each assay tube to resuspend the bead pellet. Flow cytometric data was acquired using the BD AccuriC6 Sampler counting 2100 gated events. This ensures that the sample file contains approximately 300 events per Capture Bead. Data analysis was performed using the FCAP Array analysis software.

### Genomic DNA extraction

Genomic DNA was extracted from whole blood samples of the study subjects (80 normotensive and 60 PE). Cells were transferred to 600μL lysis buffer (0.5% SDS, 150mM NaCl, 10mM EDTA, 10mM Tris-HCl (pH 8.0)). To this, RNase A (100μg/mL; DNase free) was added to the solution and incubated (37°C, 1h). Proteinase K (200μg/mL) was then added and incubated (50 °C, 3h). Protein contaminants were then precipitated by adding 5mM 0.1% potassium acetate before centrifugation (5,000xg; 15min). Supernatants containing genomic DNA were transferred to fresh tubes and extracted with 100% isopropanol on ice, and thereafter washed with 70% ethanol. DNA samples were dissolved in 10mM Tris and 0.1mM EDTA (pH 7.4, 4 °C). DNA concentration was determined using the Nanodrop2000 spectrophotometer, and all samples were standardised to a concentration of 10ng/μL.

### Genotyping

An optimised PCR was used to obtain the highest specificity and yield of the 147bp PCR product. This was achieved by amplification of the genomic DNA using 40*p*mol of each primer (Forward Primer: 5’-CATGGGTTGTGTCAGTGTCAGAGCT-3’; Reverse Primer: 5’-TGCCTTCTGTCTCCAGTCTTCCAA-3’). A no-template sample was run with the positive samples as a quality control measure against PCR contamination. The 30μL reaction consisted of 200mM of each dNTP, 2.5mM MgCl_2_, 1× Green GoTaq Flexi buffer, 0.2U Go-Taq DNA polymerase (Promega) and 30ng genomic DNA template. PCR was performed under the following cycling conditions: 94 °C for 10min (initial denaturation), followed by 30 cycles of 94 °C for 30s, 65 °C for 30s (annealing) and 72 °C for 7min (final extension). PCR products were electrophoresed on agarose gel (1.8%) and visualised using the Uvitech image documentation system (Uvitech Alliance 2.7).

**Fig. 1:**
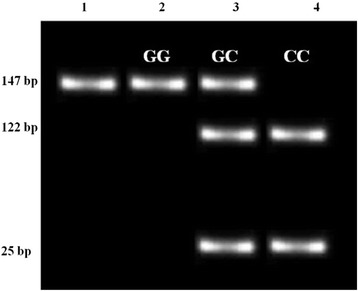
Diagrammatic representation of PCR-RFLP product sizes and corresponding genotypes. Lane 1 represents the PCR product

PCR–RFLP was used to determine the miR-146a rs2910164 genotypes. Briefly, 15μL of each PCR product was subjected to digestion by 1.5μL (10u/μL) *Sac* I and 2μL 10x Buffer-*Sac* I (Fermentas). Overnight digestion occurred at 37 °C, and thereafter digested products were electrophoresed on agarose gel (3%) and visualised as was the PCR product. Presence of the wild-type G-allele resulted in no cleavage of the PCR product. The variant C-allele yielded two fragments of 122 and 25bp. The homozygous genotype yielded three bands of 147, 122 and 25 bp. Restriction products were run alongside a DNA ladder for accurate reading of fragment sizes, thus enabling correct analysis of genotypes (Fig. 1).

### Statistical analysis

Statistical analysis was done using GraphPad Prism 5.0. Correlation between continuous variables was assessed using the Spearman rank correlation coefficient. Comparisons of mean across 3 or more groups were done using the oneway ANOVA and Ad-hoc Kruskal–Wallis test. The Pearson Chi-square (χ^2^) test was used to test association between group(s) and categorical explanatory variables. The Hardy–Weinberg equilibrium (HWE) was used to test for deviation of allele/genotype frequency. Allele and genotype frequencies were calculated using the Fisher’s exact and Chi square tests, respectively. Severe PE and early-onset PE were tested using the Barnard’s Test using an online calculator (SciStatCalc, 2013: http://scistatcalc.blogspot.co.za/2013/11/barnards-test-calculator.html; Author: Alijah Ahmed, Date Accessed: 01 December 2016). In the determination of significance, a *p*-value < 0.05 was deemed statistically significant.

## Results

The clinical characteristics of participants are shown in Table 1. The study cohort was divided into four subgroups groups:

**Table 1:** Clinical characteristics of participants

Variable	PE Total (*n* = 97)	Normo Total (*n* = 95)	PE HIV- (*n* = 53)	PE HIV+ (*n* = 44)	Normo HIV– (*n* = 50)	Normo HIV+ (*n* = 45)	*p* value
Mean age (mean ± SEM)	26.6 ± 0.668	26.2 ± 0.673	24.8 ± 0.723	28.7 ± 1.108	24.6 ± 0.904	28.0 ± 0.947	0.708^a^ 0.002 ^b, d, f^ 0.6081^c^
Age range (years)	16–42	16–46	16–40	16–42	16–42	17–46	
Parity n (%)							
0	44 (45)	22 (23)	26 (49)	18 (41)	17 (34)	5 (11)	0.006^b^
1–5	53 (55)	70 (74)	27 (50)	26 (59)	31 (62)	39 (87)	0.400^c^
> 5	1 (1.1)	3 (3.2)	0 (0.0)	1 (2.2)	2 (4.0)	1 (2.2)	
Mean CD4 (x10^6^/l) (n)	447 ± 28.2 (44)	427 ± 31.1 (47)	670 ± 154 (2)	437 ± 28.0 (42)	399 ± 66.9 (7)	432 ± 34.9 (40)	0.6346^a^ 0.431^b^ 0.9212^c^
BMI (n)	31.5 ± 0.847 (73)	30.0 ± 0.889 (71)	30.4 ± 1.30 (35)	32.5 ± 1.10 (38)	29.8 ± 1.22 (35)	30.2 ± 1.30 (36)	0.2126^a^ 0.2148^b^ 0.179^c^
MOD n (%)							<0.001^b^
ELCS	18 (19)	52 (55)	9 (17)	9 (20)	25 (50)	27 (60)	<0.001^c^
EMCS	55 (56)	24 (25)	32 (60)	23 (52)	13 (26)	11 (24)	
NVD	24 (25)	15 (16)	12 (23)	12 (27)	9 (18)	6 (13)	
TOTAL	97 (100)	91 (96)	53 (100)	44 (100)	47 (94)	44 (98)	
Blood Pressure (n)							
- Systolic	158 ± 1.75 (86)	119 ± 1.32 (62)	157 ± 17.2 (50)	159 ± 2.17 *N* = 43	119 ± 8.81 (29)	119 ± 2.03 (33)	<.0.0001^a^ <.0.0001^b, e, f, g, h^ <.0.0001^c^
- Diastolic	103 ± 1.24 (93)	74.7 ± 1.11 (62)	102 ± 13.5 (50)	104 ± 1.51 (43)	73.9 ± 8.76 (29)	75.3 ± 1.53 (33)	<.0.0001^a^ <.0.0001^b, e, f, g, h^ <.0.0001^c^
Severe PE (%)			18 (34)	21 (48)			0.121^b^
EOPE (%)			14 (26)	18 (40)			0.143^b^
Cytokines							
IL-2 (n)	265 ± 3.57 (17)	270 ± 0.949 (59)	273 ± 4.76 (7)	260 ± 4.59 (10)	274 ± 1.24 (29)	267 ± 1.21 (30)	0.1827^a^ 0.006^b, d, f^ 0.164 ^c^
IL-4 (n)	251 ± 5.40 (18)	244 ± 1.90 (59)	258 ± 26.7 (8)	245 ± 5.97 (10)	248 ± 16.4 (29)	241 ± 2.14 (30)	0.2537^a^ 0.0579^b^ 0.4760^c^
IL-6 (n)	150 ± 8.28 (18)	152 ± 4.29 (59)	174 ± 36.5 (7)	136 ± 7.90 (11)	154 ± 28.1 (29)	150 ± 6.83 (30)	0.8938^a^ 0.0057^b^ 0.1920^c^
IL-10 (n)	138 ± 46.0 (16)	81.7 ± 10.2 (54)	237 ± 251 (7)	61.4 ± 10.5 (9)	95.9 ± 68.0 (29)	66.4 ± 15.8 (26)	0.249^a^ 0.085^b^
IL-17a (n)	1520 ± 311 (16)	800 ± 31.5 (58)	785 ± 118 (8)	2250 ± 509 (8)	773 ± 36.2 (29)	827 ± 62.9 (29)	0.036^a^ <0.0001^b, f, h, i^ 0.027^c^
TNF-α (n)	186 ± 12.4 (19)	175 ± 4.01 (60)	203 ± 72.7 (8)	173 ± 10.3 (11)	186 ± 15.6 (29)	164 ± 6.78 (31)	0.416^a^ 0.0008^b, d, g^ 0.479^c^
IFN-γ (n)	214 ± 11.1 (18)	281 ± 64.7 (60)	221 ± 70.5 (8)	209 ± 4.82 (10)	221 ± 15.4 (29)	336 ± 125 (31)	0.317^a^ 0.036^b^ 0.319^c^

Means represented as mean ± SEM (n)

*Abbreviations*: *GA* gestational age, *BMI* Body Mass Index, *MOD* mode of delivery, *ELCS* elective caesarean section, *EMCS* emergency caesarean section, *NVD* normal vaginal delivery, *SEM* standard error of mean, *PE* preeclampsia, *NORMO* normotensive, *n* total number, *p* is significant at <0.05

^a^Comparison between NORMO and PE

^**b**^Comparison amongst all four sub-groups (or between PE HIV- vs PE HIV+ for Severe PE and EOPE)

^**c**^Comparison between PE HIV+ and NORMO HIV+

^d^Ad-hoc test between Normo HIV – and Normo HIV +

^e^Ad-hoc test between Normo HIV – and PE HIV -

^f^Ad-hoc test between Normo HIV – and PE HIV +

^g^Ad-hoc test between Normo HIV + and PE HIV -

^h^Ad-hoc test between Normo HIV + and PE HIV +

^i^Ad-hoc test between PE HIV – and PE HIV +

1. HIV-uninfected preeclamptic women (PE HIV-)
2. HIV-infected preeclamptic women (PE HIV+)
3. HIV-uninfected normotensive women (Normo HIV-)
4. HIV-infected normotensive women (Normo HIV+)

All women were in the third trimester of pregnancy and the mean gestational age was 36.5 weeks of pregnancy. There was a significant difference in the parity across all groups (*p* = 0.006) but not between the HIV-positive pre-eclamptic (PE) women and the normotensive group (*p* = *0.400*). There was also a significant difference in age across the groups (*p* = 0.002), and a significant difference in both the systolic and diastolic blood pressures between the total PE and normotensive groups, between all four sub-groups, and between PE HIV-positive women and normotensive HIV-positive women (all *p* < 0.0001). Only IL-2, IL-17a, TNF-α and IFN-γ showed significant differences between all four sub-groups (respectively, *p =* 0.006; < 0.0001; 0.0008; 0.036), and only IL-17a was significantly higher in the pre-eclamptic group (*p* = 0.036) The average duration of HAART was 16.6 and 14.5 weeks in the normotensive and pre-eclamptic group respectively, however this is not a precise duration of exposure. There were differences in the mode of delivery; however these were based on obstetric related indications. CD4 counts were not routinely performed on uninfected women.

The genotype and allele frequencies are shown in Table 2. There were no significant differences between the GC, CC and GG genotypes (*p* = 0.430) or G/C allele frequencies (*p* = 0.707) when compared between all preeclamptic and all normotensive women. The genotype distribution was compatible with the HWE in the study sample for both the PE and normotensive groups (*p* = 0.627; *p* = 390).

**Table 2:** miR-146a genotype and allele frequency distribution in Normotensive and PE women

	Normotensive (*n* = 80)	Pre-eclamptic (*n* =60)	*p*-value (Odds ratio; 95% CI)
Genotype n (%)			
GG	31 (38.8)	23 (38.3)	0.430
GC	38 (47.5)	24 (40.0)	
CC	11 (13.8)	13 (21.7)	
Allele n (%)			
G	100 (62.5)	70 (58.3)	0.707 (1.119; 0.6843–1.828)
C	60 (37.5)	50 (41.7)	
HWE *p*-value	0.627	0.390	

*Abbreviations*: *HWE* Hardy Weinberg Equilibrium, *CI* confidence interval, *G* guanine, *C* cytosine

Table 3 represents a sub-analysis of the genotype and allele frequencies among women stratified according to HIV status (i.e. negative or positive). No significant differences were noted in the genotype or allele frequencies by group comparison.

**Table 3:** Genotype and allele frequencies between groups

	NORMO HIV- (*n* = 42) (%)	NORMO HIV+ (*n* =38) (%)	PE HIV- (*n* =31) (%)	PE HIV+ (*n* =29) (%)
Genotype n (%)				
GG	17 (40.5)	14 (36.8)	13 (41.9)	10 (34.5)
GC	20 (47.6)	18 (47.4)	12 (38.7)	12 (41.4)
CC	5 (11.9)	6 (15.8)	6 (19.4)	7 (24.1)
Allele n (%)				
G	54 (64.3)	46 (60.5)	38 (61.3)	32 (55.2)
C	30 (35.7)	30 (39.5)	24 (38.7)	26 (44.8)
HWE	0.971	0.999	0.591	
Group comparison:	Genotypes		Alleles (G/C)	
	*p*-values		*p*-values (OR; CI)	
Norm HIV- vs. Norm HIV+	0.2420		0.6284	
			(1.174; 0.6183–2.229)	
Norm HIV- vs. PE HIV-	0.6098		0.7316	
			(1.137; 0.5767–2.241)	
Norm HIV+ vs. PE HIV+	0.4661		0.5973	
			(1.101; 0.8105–1.495)	
PE HIV—vs. PE HIV +	0.8179		0.5793	
			(1.286; 0.6215–2.663)	
Comparison between all four groups	0.6734		0.7516	

Table 4 represents the *p* values for the cytokines and selected features associated with PE. There were no significance differences noted with any of the mean cytokine levels or the other variables assessed in the PE group and sub-groups, except for severe preeclampsia, where the variant (GC/CC) genotypes were associated with significantly lower incidences of severe PE in both the total PE group (*p* = 0.0497) and the PE HIV+ group (*p* = 0.017). In the normotensive group, the variant genotypes were associated with lower IL-2 in both the total normotensive group (269 ± 1.26 (36) vs 273 ± 1.31 (23); *p* = 0.035) and the PE HIV+ sub-group 265 ± 1.54 (19) vs 271 ± 1.38 (11); *p =* 0.008).

**Table 4:** Clinical parameters analysed per genotype for pre-eclampsia and normotensive groups

	GC/CC vs. GG^a^		
	PE Total	PE -	PE +
Age	0.869	0.320	0.950
	26.5 ± 1.11 (37) vs 26.8 ± 1.14 (36)	24.3 ± 1.28 (18) vs 26.0 ± 1.08 (26)	28.6 ± 1.68 (19) vs 28.8 ± 3.04 (10)
BMI	0.675	0.225	0.390
	(31.7 ± 1.25 (29) vs 32.6 ± 1.92 (17))	(29.6 ± 1.66 (14) vs 34.4 ± 3.29 (8))	33.6 ± 1.77 (15) vs 31.1 ± 2.20 (9)
CD4	0.121	-	0.213
	(414 ± 38.1 (20) vs 538 ± 65.0 (10))		408 ± 39.8 (19) vs 506 ± 63.4 (9)
SYS BP	0.866	0.875	0.671
	160 ± 2.32 (45) vs 160 ± 3.91 (12)	158 ± 3.26 (23) vs 157 ± 8.69 (5)	161 ± 3.37 (22) vs 163 ± 3.04 (7)
DIA BP	0.817	0.654	0.412
	104 ± 1.57 (45) vs 105 ± 3.16 (12)	102 ± 2.03 (23) vs 99.4 ± 6.04 (5)	105 ± 2.43 (22) vs 108 ± 2.91 (7)
Severe PE (%)	0.0497	0.364	0.017
	14 (38) vs 14 (61)	7 (39) vs 6 (46)	7 (37) vs 8 (80)
EOPE (%)	0.497	0.501	0.498
	14 (38) vs 9 (39)	5 (28) vs 4 (31)	9 (47) vs 5 (50)
IL-2	0.201	0.240	0.885
	262 ± 4.78 (10) vs 282 ± 13.1 (7)	268 ± 6.83 (4) vs 279 ± 5.09 (3)	258 ± 6.54 (6) vs 260 ± 7.54 (3)
IL-4	0.758	0.801	0.549
	(247 ± 8.18 (10) vs 251 ± 5.29 (7))	261 ± 18.9 (4) vs 255 ± 7.34 (4)	238 ± 4.12 (6) vs 244 ± 7.08 (3)
IL-6	0.095	0.534	0.334
	139 ± 7.93 (10) vs 171 ± 15.5 (7)	163 ± 14.5 (3) vs 181 ± 22.7 (4)	128 ± 6.66 (7) vs 158 ± 22.3 (3)
IL-10	0.895	0.697	0.107
	147 ± 70.3 (9) vs 133 ± 70.4 (6)	273 ± 140 (4) vs 188 ± 147 (3)	45.4 ± 15.8 (5) vs 78.5 ± 2.77 (3)
IL-17a	0.758	0.475	0.511
	1430 ± 313 (9) vs 1690 ± 728 (6)	820 ± 32.1 (4) vs 750 ± 79.3 (4)	1920 ± 466 (5) vs 3550 ± 1630 (2)
TNF-α	0.920	0.418	0.316
	186 ± 15.4 (11) vs 183 ± 25.2 (7)	227 ± 25.4 (4) vs 179 ± 45.5 (4)	164 ± 14.0 (7) vs 189 ± 16.9 (3)
IFN-γ	0.315	0.507	0.668
	206 ± 17.0 (11) vs 227 ± 11.2 (6)	201 ± 50.4 (4) vs 240 ± 10.6 (4)	208 ± 5.02 (7) vs 200 ± 12.6 (2)

Mean ± SEM (n)

*Abbreviations*: *BMI* body mass index (kg/m^2^), *EOPE* early onset preeclampsia, *SYS* systolic, *DIA* diastolic, *BP* blood pressure (mmHg), *IL* interleukin, *TNF*-α tumour necrosis factor alpha, *IFN*-γ interferon gamma

^a^ *p* value

## Discussion

The dysregulation of miRNA146a has been associated with inflammatory diseases such as RA and SLE [22, 25]. A downregulation of miRNA 146a has been found in placentas of preeclamptic women compared with normal women [26], therefore suggesting a potential role for miRNA146a in the pathogenesis of preeclampsia. Based on the presence of an increased inflammatory environment in preeclampsia [27–29], polymorphisms in the miRNA146a gene may therefore affect susceptibility to preeclampsia. The rs2910164 G > C polymorphism of miRNA146a has recently been studied in inflammatory conditions [25], however its role in preeclampsia and co-morbid HIV infection has not been evaluated.

Inflammation is mediated by a variety of soluble factors, including cytokines [30], which were evaluated in this study. Preeclampsia is characterised by significantly higher levels of pro-inflammatory cytokines such as IL-6 and TNF-α, when compared with normal pregnant women [31]. However, according to Celik et al. 2012, studies on circulating interleukins are inconsistent [10]. This could be attributed to the fact that cytokines have a short half-life and transient, episodic release [11], which may even differ at different gestational periods, or the fact that endothelial sensitivity to cytokines may differ among women, rendering normal levels pathological [11]. A speculation could be that SNPs could be involved in cytokine dysregulation, resulting in the inconsistencies noted. In HIV infection, the move away from a pro-inflammatory cytokine milieu that occurs as disease progresses, is counteracted with the usage of HAART [32].

Our data did not show any significant association in the frequency of the variant genotypes (GC/CC) in women with preeclampsia and there was no significant association in the groups stratified according to HIV. Furthermore, we did not find a significant relationship of the variant genotypes with cytokines or the features of preeclampsia that were assessed when stratified according to HIV status. This could be due to the influence of the SNP on miR-146a levels, and consequent expression of cytokines. However, it was found that IL-2, a pro-inflammatory cytokine of the Th1 response, was significantly lower in the variant genotypes for both the total normotensive group, and the stratified HIV+ group, indicating a possible role of the SNP in reducing the pro-inflammatory response.

Another significant finding was that the incidence of severe PE was significantly lower for the variant genotype in the PE total and stratified PE HIV+ group, suggesting a reduced susceptibility for the development of severe disease with the variant genotype, and a differential dysregulation in the presence of HIV infection.

Severe PE is associated with adverse neonatal outcomes, is more likely to recur [33], and is also associated with increased maternal morbidity [34]. Moreover, women with severe PE are at high risk for cardiovascular disease later in life [35]. Eclamptic seizures, intracerebral haemorrhage, pulmonary oedema or heart failure, acute renal failure, liver dysfunction, and coagulation abnormalities are all associated with severe pre-eclampsia [36]. Foetal complications include intrauterine growth restriction, premature delivery, and intrauterine foetal death [36]. Eclampsia, a severe form of PE characterised by seizures, is associated with a (0%-1.8%) mortality rate in developed countries rising to a high rate of 15% in developing countries [37]. Furthermore, obesity is a known risk factor for PE [38], a finding relevant to our study (mean BMI among the HIV infected preeclamptic women was 32.5 kg/m^2^).

The role of miRNA-146a in HIV infection has emerged more recently. Spinello et al. reported a relationship between miR146a and CXCR4 co-receptor in HIV [39]. Resting CD4 + T cells have high expression of miR-146a, which inhibits the expression of the co-receptor CXCR4, and prevents the HIV entry in CD4 + T cells [39, 40]. Furthermore, Duskova et al. [41] demonstrated that miR-146a was significantly increased in infected patients as compared to healthy controls.

Our study demonstrates the relationship of miR-146a rs2910164 G > C polymorphism with PE, some of the related features, cytokines and HIV/HAART. Of note is the increased susceptibility to severe disease in local women with the ancestral genotype, and reduced pro-inflammatory circulating IL-2 levels in the normotensive women with the variant genotypes.

### Study Limitations

Our findings are preliminary and limited by a small sample size. Further, the influence of other functional polymorphisms on miR-146a expression in co-morbidities needs further investigation, and the expression of miR-146a needs to be measured. A larger cohort will enable further research. This study was restricted to SA Black women, thus our findings may not necessarily apply to other ethnic groups.

## Conclusion

MiR-146a rs2910164 G > C polymorphism might not be associated with PE susceptibility, cytokines or related features. However, the miR-146a GC/CC variant genotypes might reduce susceptibility to severe PE, which might be further influenced by the presence of co-morbid HIV infection among pregnant women on HAART.

## Abbreviations

AHT: Antihypertensive drugs
BMI: Body mass index
CI: Confidence interval
DIA BP: Diastolic blood pressure
ELCS: Elective caesarean section
EMCS: Emergency caesarean section
EOPE: Early onset preeclampsia
GA: Gestational age
HAART: Highly active antiretroviral therapy SA, South African
HWE: Hardy–Weinberg equilibrium
IRAK-1: Interleukin-1 receptor associated kinase 1
miRNA/miR: MicroRNA
MOD: Mode of delivery
NORMO: normotensive
NVD: Normal vaginal delivery
OR: Odds ratio
PCR-RFLP: Restriction fragment length polymorphism
PE: Preeclampsia
RA: Rheumatoid arthritis
SD: Standard deviation
SLE: Systemic lupus erythematosus
SNP: Single nucleotide polymorphism
SYS BP: Systolic blood pressure
TRAF-6: TNF receptor associated factor
